# Do system quality and information quality affect job performance? The mediation role of users’ perceptions

**DOI:** 10.1371/journal.pone.0285293

**Published:** 2023-06-23

**Authors:** Deddy Eduar Eka Saputra, Vera Maulida Rahma, Anis Eliyana, Andika Setia Pratama, Rachmawati Dewi Anggraini, Nurul Liyana Mohd Kamil, Izlin Ismail

**Affiliations:** 1 Postgraduate School, Universitas Negeri Jakarta, East Jakarta, DKI Jakarta, Indonesia; 2 Directorate General of Corrections, Ministry of Law and Human Rights of the Republic of Indonesia, Central Jakarta, DKI Jakarta, Indonesia; 3 Department of Management, Universitas Airlangga, Surabaya, East Java, Indonesia; 4 Research and Publication, PT Usaha Mulia Digital Indonesia (PT UMDI), South Jakarta, DKI Jakarta, Indonesia; 5 Department of Political Science, Public Administration and Development Studies, Universiti Malaya, Kuala Lumpur, Malaysia; 6 Department of Finance, Universiti Malaya, Kuala Lumpur, Malaysia; COMSATS University Islamabad - Wah Campus, PAKISTAN

## Abstract

This study aims to analyze the influence of the system quality and information quality from the Correctional Database System (CDS) on the job performance of correctional Technical Support Officers (TSO) in Sumatra, either directly or through the mediating role of perceived ease of use and perceived usefulness. This study tested 118 correctional officers selected by the purposive sampling technique. A quantitative approach is used with Partial Least Square–Structural Equation Modeling analysis technique. It was found that the direct influence of the system and information quality on job performance is insignificant. Nonetheless, it shows that perceived ease of use and perceived usefulness fully mediate system quality and information quality on job performance. The findings demonstrate that perceived ease of use and perceived usefulness are important factors that motivate individual performance when supported by high-quality information systems. This study provides an overview of the use of a mandatory system for public organizations and the effects on user performance. It can be used as a basis for correctional institutions to strengthen and maintain a positive perception of CDS among their officers, such as through mentoring programs.

## Introduction

The public has long scrutinized the work of government authorities in the sphere of services such as Correctional Institutions, which in Indonesia legal system refers to a facility in which offenders serve their sentences following a court verdict. It is evidenced by the number of complaints from the general public, particularly regarding the still convoluted (bureaucratic) service systems and procedures, the uncertain service period, the unclear, ambiguous service information, and the attitude and behavior of the officers in providing services. With the growth of information technology, the service system may be computerized, allowing for public services improvement and administrative problems elimination. Information technology is crucial because it may help people be more focused and operate more efficiently, eventually enhancing their productivity and performance. Since 2013, correctional institutions have utilized the Correctional Database System (CDS) as a tool for officers’ work in their operational tasks. CDS is a system that belongs to the Directorate General of Corrections in the Republic of Indonesia. It is a reporting mechanism and unified data management system for correctional institutions that also serves as a work assistance [1]. It involves collecting, filtering, managing, presenting, and transmitting Correctional information.

The introduction of CDS is a digital transformation; a set of actions carried out by organizations in response to changes by modifying operational activities using digital technology [2]. The digital transformation implemented by correctional institutions is a response to the ever-increasing pace of technological advancement, as organizations that are properly digitalized will increase job effectiveness, productivity, employee engagement, and revenue growth [3]. In public organizations, the use of digital technologies can improve service efficiency and quality by decreasing service waiting times, enhancing transparency, and providing seamless service delivery across the organization [4].

It is done because officers must maintain excellent performance to give excellent service, even though correctional facilities have few human resources compared to the number of residents who must be served. Given the features of Sumatra Island, a border island with a high level of susceptibility, the ratio of officers to prisoners in Sumatra’s correctional facilities is 1:11. Even in 2020, the number of residents increased by 19 percent, while the number of officers declined by 14.5 percent. This phenomenon produces a backlog of work and increased expectations for correctional services on Sumatra, particularly for the correctional TSO, whose jobs include Finance, Personnel, Equipment, General, Administrative, and General Functional. A correctional institution officer in Sumatera can use CDS to perform service duties such as administering registrations, human resources, finances, and data consolidation. CDS maintains and reports comprehensive statistics on each prisoner during their confinement. This can rectify the ineffectiveness and inefficiency of data processing in the past, which frequently hindered the fulfillment of prisoners’ rights.

In this instance, CDS aims to increase performance to compensate for correctional institutions’ paucity of human resources, since individual with high performance leads to an increase in overall institutional performance [5]. Due to insufficient TSO in Sumatra and the community’s negative service evaluations, officers need help with overtime. The digital transformation of Sumatra’s prisons is still in progress, despite the CDS’s value in preserving data about correctional facilities to assist in decision-making. According to data from September 2021, 47 percent of Sumatera’s correctional facilities did not fully update or disclose their CDS data, indicating that CDS was not optimally utilized. Sumatra’s correctional facilities continue to be dispersed and isolated, making technological reforms more difficult. Socialization and assistance in regards to the use of CDS are examples of possible actions. On the other side, the absence of usage of CDS in Sumatran correctional institutions, which was initially intended to aid officers’ jobs, and its influence on performance have yet to be intensively investigated.

Job Performance (JP) is a collection of activities, behaviors, and job outcomes that may be quantified and contribute to achieving corporate objectives [6]. This study assesses JP as the effect of implementing a system in government agencies. According to the Technology Acceptance Model, the usage of a system is influenced by two key criteria, namely perceived ease of use (PEOU) and perceived usefulness (PU) [7], which subsequently influence the user’s JP [8]. PEOU is the user’s perception of the amount to which using the system requires little or no effort, whereas PU is the user’s perception of the extent to which using the system provides advantages to its users [7].

Moreover, JP is also affected by the processing quality of the system itself, or what is usually referred to as system quality (SQ) and information quality (IQ), or the quality of the information provided by a system for its users [9, 10]. In the context of the job of the Sumatran TSO, SQ refers to the success of entering registration data and resident fingerprints, applying for remissions, and gaining access to all data and features. In addition, the questioned IQ is related to the appropriateness of personal data and occupant records and the compatibility of the number of occupants and room capacity to the actual situation.

Numerous studies on the impact of information systems on performance have been uncovered so far, but nearly fewer have been undertaken in Correctional Institutions. In addition, although some research has explored the mediating impact of PEOU and PU on the influence of SQ and IQ on JP, the results have been inconsistent. To address these limitations, the primary purpose of this study is to determine the effect of the Correctional Database System (CDS) on the performance of Technical Support Officers in Sumatra’s correctional facilities. In addition, the results of this study will also contribute to the management of the Directorate General of Corrections in the Republic of Indonesia, especially for correctional units in Sumatra where the digital transformation process is still on going.

In light of the theoretical foundation and literature review, this study developed the hypothesis that the system quality and information quality of HR influence the job performance of correctional Technical Support Officers in Sumatra, either directly or indirectly, via perceived ease of use and perceived usefulness. All hypotheses that have been compiled will be examined using Partial Least Squares–Structural Equation Modelling (PLS-SEM). This study concludes by discussing the research’s implications, limitations, and suggestions for academic and correctional institutions in Sumatra.

## Literature review

Technology Acceptance Model shows that the organization’s digitization process must be balanced with human resource management, who will later become system users [7]. Technology adoption must be prioritized for an organization since it has a long-term impact on how the organization survive. For example, how these technologies are used will depend on how well employees can adapt to new technologies and respond to them [1]. How users receive and utilize a system is based on PEOU or the degree to which they believe that using a system will require little or no effort [11]. Whereas PU is the extent to which a person believes that the use of a system can help them work and improve their job performance [12]. According to a prior study, adoption readiness has a substantial direct impact on attitude toward using CDS. Adoption readiness is thought to have a significant impact on how individuals view using the system and adjusting to it. It is closely tied to personality traits and emotional responses. Also, it can help user technology acceptance, which also affects satisfaction, and help individual’s positive attitudes toward using technology [1]. A previous study stated that the indicators of technology readiness that affect PEOU and PU users in using a required system, like CDS, are optimism and innovativeness [13]. The two officers’ perceptions are not only determined by technology readiness owned by officers in the system acceptance process, but there are external factors that also play a role in forming the PEOU and PU. These factors relate to the nature of the system itself, which is related to the SQ and IQ they receive.

The Information System (IS) Success Model then goes on to explain that a taxonomy of numerous variables can be used to evaluate the success of an IS [14]. Six interconnected IS-related aspects are included in the taxonomy: system quality (SQ), information quality (IQ), use (using a system), user satisfaction (user satisfaction with using a system), individual impact (impact of using a system on an individual), organization impact (impact of using a system on an organization). SQ measures how well the system is technically operating and takes into account the software and data used [15]. IQ is the degree to which users can use a system to receive accurate, timely, relevant, and complete information while also making their work easier [12]. According to this justification, the effectiveness of IS can be judged by how it is used, how satisfied users are with it, and how SQ and IQ affect people and organizations. In keeping with that, CDS aims to create a national database of correctional institutions, so it is crucial that the information management process runs smoothly from gathering to communicating data from all work units. In addition, IQ is one of the key areas that can support decision-making and enhance officer services, following the goal of creating the CDS.

So far, many studies have found that examine the direct effect of SQ and IQ on JP, as well as indirect influence through the mediating role of PEOU and PU. The results could have been more consistent from many previous studies on the role of mediation. In previous research, it was found that PEOU and PU were able to fully mediate the effect of SQ and IQ on JP [13], some stated that mediation was partial [6, 7], and it was also found that the two variables had not been able to mediate [14]. By referring to the existing phenomena, this study aims to explore how the use of CDS by TSO in Sumatran correctional institutions relates to the quality of the system and the quality of information, as well as the role of officers’ perceptions of the ease and usefulness of CDS, to their effect on the performance of the Sumatran correctional TSO.

So far, although several studies have discussed the effect of the use of information systems on performance, not much has been done in the context of Correctional Institutions. Furthermore, research that examines the mediating role of PEOU and PU on the effect of SQ and IQ on JP, shows inconsistent results. In previous research, it was found that PEOU and PU were able to fully mediate the effect of SQ and IQ on JP [16], however some studies found that the mediation was partial [9, 10] and the other even found no mediation effect [17]. To overcome these limitations, the main objective of this research is to find out the role of CDS on TSO correctional performance.

### Hypothesis development

#### System quality and job performance

One study in the context of university employees found that SQ have a significant effect on JP [17]. This relation has also previously been proven by a study stating that there is a significant direct effect of SQ on users’ performance of employees in Tunisia, in which the highest SQ indicator is system integration and reliability [9]. A system that successfully integrates information across all lines, and can manage that information well at all times, will encourage the performance of its users. SQ is also considered an important aspect that contributes to JP in the context of individual performance users when using Enterprise Resource Planning systems (ERP). Hence, managers must ensure ERP users are satisfied with SQ [18].

**H1: System Quality has a significant influence on Job Performance**

#### Information quality and job performance

According to the previous study, Tunisia has a sizable direct effect of IQ on user performance [9]. Completeness and recency make the biggest impact on how well a user perform when it comes to IQ. When users have access to complete information that helps them complete their tasks and reach their objectives, the level of performance improves. IQ is a crucial factor that influences users’ performance because accuracy, relevance, and the availability of information that they need can minimize errors and increase the efficiency of their work [9]. This finding is also supported by a previous study [10], which found that IQ directly influences the performance of medical officers at the UNRWA-Gaza health center.

**H2: Information Quality has a significant influence on Job Performance**

#### Perceived ease of use and job performance

In the context of the impact on individual performance, prior research made PEOU one component of forming the JP [16]. Due to the increase in the JP system, the users can allocate their effort to other tasks, making their work more effective and efficient. A system with a high PEOU rate will minimize the effort given by the user to be able to run it [7]. Further research established that one factor influencing performance is the PEOU held by senior librarians in Pakistani university libraries opposing the use of big data analytics [8]. The ease of accessing and using the system will have an impact on senior librarians’ performance as users when jobs change in accordance with technology.

**H3: Perceived Ease of Use has a significant influence on Job Performance**

#### Perceived usefulness and job performance

Previous research found that JP can be impacted by individual factors like knowledge and technological factors like PU owned by employees [19]. Additionally, a different study demonstrates that one of the factors influencing performance is the senior librarians’ PU in Pakistani university libraries on the use of big data analytics [8]. Additionally, managing and storing research data is a new duty for librarians. To support higher-level research, libraries also need to gather, arrange, and access data or information. Therefore, when big data analytics can make librarians’ jobs easier, they will think the system is valuable because it can influence how they perform.

**H4: Perceived usefulness has a significant effect on Job Performance**

#### System quality and perceived ease of use

In the development of the Technology Acceptance Model (TAM) [12], it is stated that SQ has a positive and significant effect on the PEOU of students who use e-learning systems. Past study stated that the PEOU of business school students in India is the leading use of information apps influenced by SQ [20]. Furthermore, it is also stated that of the three constructs that affect PEOU, SQ is the construct with the highest influence value. It indicates that the quality of system processing has a major impact on user perceptions of the ease of use of the system, so system managers or developers must identify the specific needs of users and provide elements that are relevant to their needs.

**H5: System Quality has a significant influence on Perceived Ease of Use**

#### Information quality and perceived ease of use

According to a prior study used to develop the Technology Acceptance Model (TAM) model, PEU students who use e-learning systems are significantly and positively influenced by IQ [12]. Students will perceive that operating a quality system requires little effort if they believe that the information it produces is intended to meet the criteria for good information, such as being clear and current. Additionally, according to a study [20], the PEOU of Indian students attending business schools is the country’s top user of IQ-influenced information apps. Users’ perception of the system’s usability is affected by their perception of the information’s usability. The app offers information that is current, comprehensive, and capable of satisfying user needs. Therefore, in order to enhance the quality of information, system managers or developers must concentrate on the information’s completeness, accuracy, and credibility.

**H6: Information Quality has a significant influence on Perceived Ease of Use**

#### System quality and perceived usefulness

Previously, it was found that SQ significantly affected PU on using e-learning systems by Gamsar University students Payam Noor [21]. The level of SQ affects students regarding the perception of the benefits provided by e-learning systems. In line with that, previous study also found that SQ positively and significantly affects the user’s PU level [22]. SQ describes the satisfaction of hospital employees with HIS functionality, so if employees feel HIS responds quickly and according to their intended use of the system, then HIS will be considered useful.

**H7: System Quality has a significant influence on Perceived Usefulness**

#### Information quality and perceived usefulness

The IQ positively and significantly affects PU students who use e-learning systems [12]. When users feel that the information produced by a quality system is intended to fulfill good information dimensions such as being relevant to their needs, appropriate format, and reliable, they will feel that the results of using the system can help them in completing their tasks or work. Furthermore, when hospital employees feel that HIS provides them with rich, up-to-date information, and can meet their expectations and needs, employees will feel comfortable using the system and further recognize that HIS is a useful tool [22].

**H8: Information Quality has a significant influence on Perceived Usefulness**

#### System quality and information quality on job performance mediated by perceived ease of use and perceived usefulness

It would be intriguing to discuss how PEOU and PU affect an IS’s success. In Tunisia, office workers’ user performance may be impacted by the quality of IS and the information it produces, either directly or indirectly through the mediating variables PEOU and PU [9]. It demonstrates that the influence of SQ and IQ on JP is partially mediated by PEOU and PU. According to the study, PEOU is the primary mediator of the relationship between IQ and JP because users’ perceptions of how simple it is to use IS will have a greater impact on how well they perform. Additionally, when IS offers high-quality information, users view it as the most practical system, which affects how well it performs.

Likewise, prior study also stated that there is a mediating role of PEOU and PU on the indirect effect of SQ and IQ on JP medical workers at UNRWA, Palestine [10]. Awareness of medical staff about the ability of the system to be useful and easy to use encourages increased performance in carrying out their duties in the hospital. The higher the quality of patient information in the system, the more medical staff feel that the system is useful and easy to use to complete their work, thus, the greater their intention to use the system and improve its performance.

In contrast to these results, another study found that PEOU and PU cannot mediate the direct influence of SQ and IQ on users’ performance in the context of university employees [17]. Although there is a partial effect mediation or partial mediation of PEOU and PU on the relationship between SQ and IQ in users’ performance in using the cyber campus system, this value is smaller than the value of the direct influence of SQ and IQ on the user performance. Meanwhile, another study proves that SQ and IQ do not directly influence JP but through PEOU or PU [16]. The mediating role of PEOU and PU in JP is full mediation because there was no direct effect of SQ and IQ on the JP of hospital employees in Kenya. The results of these studies are sufficient to support the statement that PEOU and PU mediate the relationship between SQ and IQ in JP.

**H9: Perceived Ease of Use significantly mediates the influence of System Quality on Job Performance**
**H10: Perceived Ease of Use significantly mediates the influence of Information Quality on Job Performance**
**H11: Perceived usefulness significantly mediates the influence of System Quality on Job Performance**
**H12: Perceived usefulness significantly mediates the influence of Information Quality on Job Performance**

**Fig 1:**
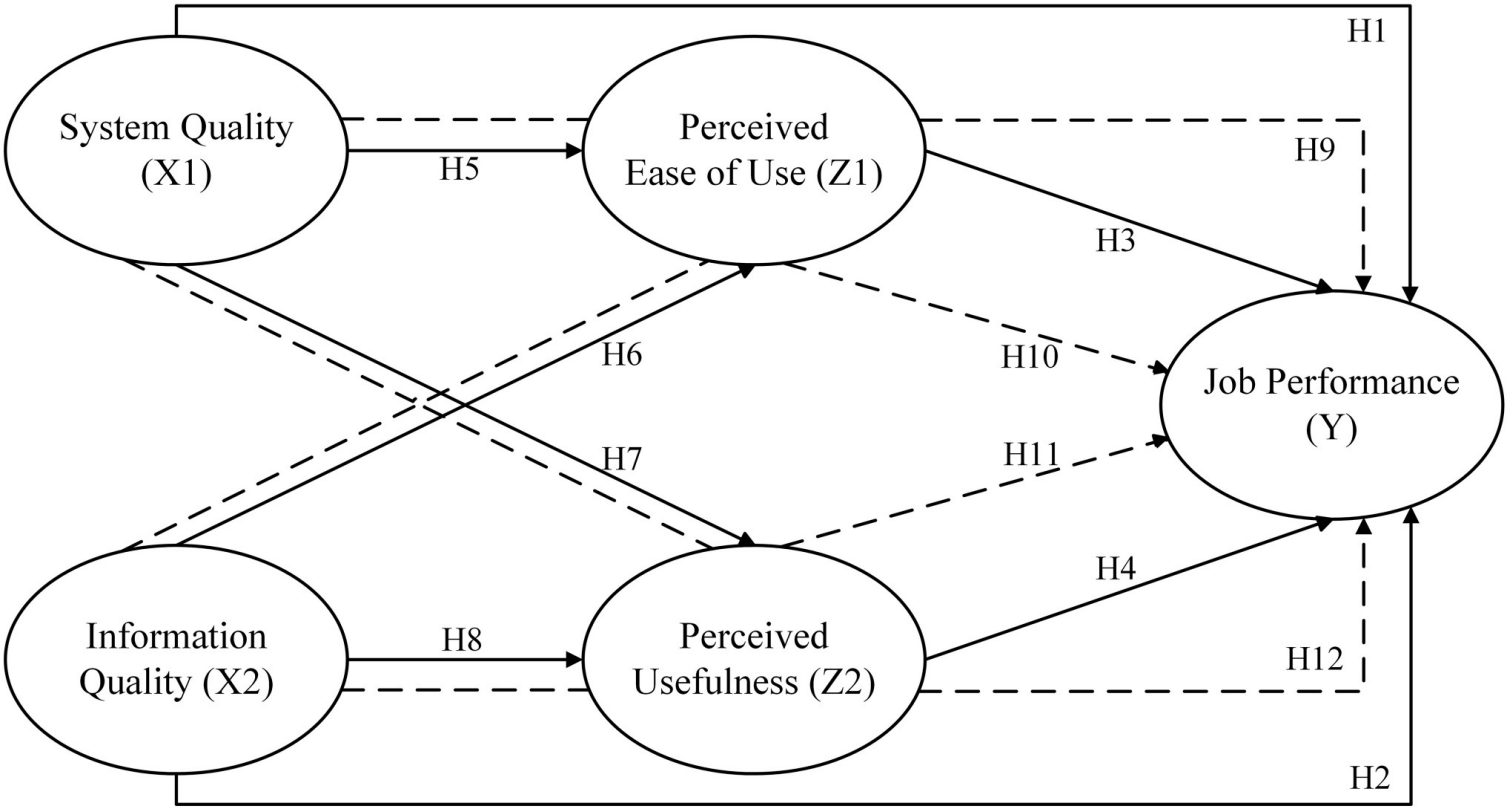
Research framework.

The overall hypotheses are conceptualized on the following framework (Fig 1).

## Research methods

### Research approach

This study uses a quantitative approach, namely a research approach that answers the research objectives through an empirical analysis and assessment approach that involves numerical measurements as statistical calculations and hypothesis testing [23]. The dependent variable in this study is Job Performance (JP), the mediating variables are Perceived Ease of Use (PEOU) and Perceived Usefulness (PU), while the independent variables are System Quality (SQ) and Information Quality (IQ). Measurements on the variables SQ, IQ, PEOU, PU, and JS used a Likert scale of 1 to 5 which will describe the correctional TSO’s perception of the existing indicators.

### Measurement

In this study, JP was measured by 9 items, such as "I can complete tasks according to the amount of work charged with CDS" [24]. SQ was measured by 5 items, such as "I am satisfied with the functioning of the CDS system" [12]. Likewise, IQ is measured by 5 items, such as "The information output of the CDS system is clear " [12]. PEOU was measured by 5 items, such as "Use of CDS is understandable" [17, 25]. Similarly, PU is measured by 6 items, such as "Using CDS makes my job easier" [7, 25]. All measurement items for each variable were originally in English and were translated into Indonesian. The measurement items of each variable were translated back into English to ensure that there was no difference in meaning when translated.

### Data collection technique

Data was collected by distributing questionnaires. The population used in this study were Technical Support Officers (TSO) in 115 correctional institutions from 10 Regional Offices in Sumatra totaling 578 officers. The sampling technique used in this research is purposive sampling, which determines the sample based on certain criteria or characteristics needed in the study [23]. The criteria are:

1. Officer who has worked for at least 3 years.
2. Officer whose work is related to CDS.
3. Officer who has an account or direct access to CDS.

The online survey was conducted during October-November 2022. As a result, there are 118 correctional officers in Sumatra who meet the criteria.

The Research Ethics Committee at Universitas Airlangga: Development and Innovation Institute for Publishing Journal and Intellectual Property Rights (LIPJIPHKI) has confirmed that no ethical approval is required. The organization’s chief has granted written informed consent on behalf of all respondents following the organization’s policy. Before completing the questionnaires, all respondents were informed that data collection was not mandatory. Their willingness to continue filling out the questionnaire signifies consent to participate. In addition, respondents were informed that their responses were guaranteed to be kept strictly confidential and used only for research purposes. LIPJIPHKI also validated the consent.

### Data analysis technique

In this study, all hypotheses that have been compiled will be tested using Partial Least Square–Structural Equation Model (PLS-SEM). In exploratory research, PLS-SEM is very valuable because it can evaluate the measurement of latent variables and test the relationship between latent variables. PLS-SEM was chosen because it can be applied to sample sizes that do not have to be large and data that do not have to be normally distributed in complex models [26]. In addition, PLS-SEM is a commonly used method to test the relationship between latent variables with little or no solid theoretical basis [26]. The steps in using the PLS-SEM analysis technique are divided into three: model specification, outer model evaluation, and inner model evaluation.

## Results and discussion

### Characteristics of respondents

Table 1 shows the characteristics of the respondents. Most respondents were male (67.8%) and were in the highest age range of 26–30 years (26.2%). They have worked for over 3 years, whereas most have even worked for 11–14 years (29.6%). All respondents are generally well educated starting from senior high school to master’s degree, where their last education was dominated by bachelor’s degree (64.4%). With these characteristics, they are assumed to be able to provide appropriate answers regarding SQ, IQ, PEOU, PU, and JP.

**Table 1:** Respondents’ demographic profiles.

Respondent Profile	Information	Frequency	Percentage
Gender	Male	80	67.8
	Female	38	32.2
Age	21–25 years old	9	7.6
	26–30 years old	31	26.2
	31–35 years old	30	25.4
	36–40 years old	28	23.7
	41–45 years old	5	4.2
	46–50 years old	7	5.9
	> 50 years old	8	6.8
Tenure	3 years	19	16.1
	4–6 Years	12	10.2
	7–10 Years	21	17.7
	11–14 Years	35	29.6
	> 15 Years	31	26.2
Highest Education Degree	Senior high school	25	21.1
	Diploma	10	8.5
	Bachelor	76	64.4
	Master	7	5.9

### Outer model test

In the outer model test, measurements will be made of the relationship between the indicator and its latent variable. The evaluation that will be carried out in this study is in the form of convergent test validity, discriminant validity, and internal consistency reliability. Convergent test validity is based on the outer value loading > 0.7 and AVE value > 0.5 [27]. Discriminant testing validity is based on the value of cross-loading. The indicator is considered valid if the value of cross loading indicator of the variable is the largest compared to other variables. Convergent Validity can be interpreted as the ability of a set of indicators to represent or underlie a latent variable. While the next step is testing internal consistency reliability. This test consists of composite reliability and Cronbach’s alpha with the provision that it must have a value of > 0.6 to be said to be reliable. Based on Table 2, the results show that all indicators have an outer. value loading > 0.7 and the AVE value > 0.5 so it can be declared valid. In addition, all variable measurements have composite value reliability and Cronbach’s alpha > 0.60 so they can be declared reliable. Thus, all measurement items can be declared valid and reliable for further analysis.

**Table 2:** Outer model test results.

Variable	Indicator	Convergent Validity		Discriminant Validity (Cross loading)					Internal Consistency Reliability	
		OL	AVE	X1	X2	Z1	Z2	Y	Cron. Alpha	CR
System Quality (X1)	SQ1	0.739	0.639	**0.754**	0.384	0.519	0.481	0.555	0.864	0.898
	SQ2	0.771		**0.712**	0.053	0.240	0.178	0.260		
	SQ3	0.849		**0.812**	0.165	0.271	0.275	0.319		
	SQ4	0.859		**0.828**	0.258	0.224	0.323	0.336		
	SQ5	0.888		**0.880**	0.214	0.314	0.371	0.410		
Information Quality (X2)	IQ1	0.883	0.763	0.363	**0.883**	0.549	0.626	0.693	0.923	0.942
	IQ2	0.823		0.164	**0.823**	0.410	0.478	0.516		
	IQ3	0.898		0.198	**0.898**	0.434	0.570	0.577		
	IQ4	0.869		0.215	**0.869**	0.495	0.529	0.566		
	IQ5	0.893		0.349	**0.893**	0.553	0.694	0.658		
Perceived Ease of Use (Z1)	PEOU1	0.802	0.744	0.372	0.482	**0.802**	0.450	0.569	0.913	0.935
	PEOU2	0.813		0.357	0.335	**0.813**	0.393	0.539		
	PEOU3	0.903		0.360	0.536	**0.903**	0.443	0.666		
	PEOU4	0.852		0.332	0.548	**0.852**	0.479	0.668		
	PEOU5	0.935		0.434	0.508	**0.935**	0.543	0.753		
Perceived Usefulness (Z2)	PU1	0.908	0.795	0.419	0.615	0.519	**0.908**	0.779	0.948	0.959
	PU2	0.882		0.421	0.579	0.445	**0.882**	0.761		
	PU3	0.873		0.367	0.628	0.442	**0.873**	0.610		
	PU4	0.927		0.373	0.590	0.430	**0.927**	0.700		
	PU5	0.898		0.412	0.589	0.612	**0.898**	0.817		
	PU6	0.859		0.373	0.590	0.415	**0.859**	0.643		
Job Performance (Y)	JP1	0.908	0.764	0.463	0.707	0.648	0.754	**0.908**	0.961	0.967
	JP2	0.871		0.410	0.642	0.746	0.649	**0.871**		
	JP3	0.878		0.481	0.696	0.690	0.781	**0.878**		
	JP4	0.866		0.472	0.628	0.752	0.677	**0.866**		
	JP5	0.882		0.445	0.624	0.484	0.863	**0.882**		
	JP6	0.853		0.495	0.459	0.704	0.572	**0.853**		
	JP7	0.909		0.477	0.602	0.543	0.820	**0.909**		
	JP8	0.886		0.450	0.560	0.720	0.624	**0.886**		
	JP9	0.810		0.287	0.524	0.600	0.601	**0.810**		

### Inner model test

Next is the inner model evaluation stage which will test the inner model’s ability to predict the relationship between constructs. The evaluation that will be carried out in this study is in the form of coefficient assessment of determination (R^2^), cross-validated redundancy (Q^2^), the effect size (f^2^), as well as path coefficients which are then followed by hypothesis testing. R^2^is a measure of the predictive accuracy of the existing model. In addition, R^2^ also describes the variance of the influence of the independent variable on the dependent variable [27]. The accuracy of the influence of the independent variable on the dependent variable can be seen based on the range 0 to 1, where the higher the value, the more accurate the effect. Table 3 shows that the Adjusted R^2^ value on the PEOU is 0.382, meaning that the percentage of the influence of SQ and IQ on PEOU is 38.2% and is included in the weak category. The value of Adjusted R^2^ on PU is 0.504, which means that the percentage of the influence of SQ and IQ on PU is 50.4% and is included in the medium category. Furthermore, the Adjusted R^2^ value on the JP variable is 0.801, which means that the percentage of the influence of SQ, IQ, PEOU, and PU on JP is 80.1% and is included in the strong category.

**Table 3:** Inner model test results.

Variable	R ^2^ Adj	f ^2^	Q^2 _^
System Quality (X1)	-	0.035	-
Information Quality (X2)	-	0.048	-
Perceived Ease of Use (Z1)	0.382	0.429	0.286
Perceived Usefulness (Z2)	0.504	0.554	0.399
Job Performance (Y)	0.801	-	0.605

Furthermore, the magnitude of the influence on each path model is determined based on the value of Cohen’s f ^2^. The value of f2 is known by observing and measuring changes in the value of R2 when certain independent variables are removed from the model [27]. This value can describe the magnitude of the effect of the omitted variable on the dependent variable. Table 3 shows that the largest f^2^ value is in the PU (0.554), meaning that the construct that gives the largest direct contribution to JP is PU, next in order are PEOU, IQ, and SQ. Then the Q^2^ value is a measurement of predictive relevance to the inner model [27]. The dependent variable is declared to have predictive relevance if the value of Q^2^ > 0 is obtained—the smaller the difference between the predicted and original values, the larger the Q^2^ value. Table 3 shows that the three variables have a Q^2^ > 0 and it can be interpreted that the independent variable has predictive relevance to the dependent variable. Predictive relevance to the JP construct is high, meaning that the variables SQ, IQ, PEOU, and PU have great relevance in predicting TSO’s JP.

### Hypothesis test

Hypothesis testing was carried out with the PLS-SEM bootstrapping process to determine whether there was a relationship between the independent and dependent variables used in this study. The test results are based on the P-value and T-statistics of the sample used. To state that there is a significant effect between variables, it takes a T- statistics value > 1.65 (5% significance level), and the P-value must be less than 0.05 [24].

Based on the results of hypothesis testing shown in Table 4, it is known that there is no significant direct effect of SQ and IQ on JP with T- statistics value < 1.65 and P-value > 0.05 so H1 and H2 are rejected. Meanwhile, the direct influence of PEOU and PU on JP was proven significant with T—statistics > 1.65 and P-value <0.05 so H3 and H4 were accepted. Likewise, the direct effect of SQ and IQ on PEOU and PU was significant with T—statistics > 1.65 and P-value <0.05 so H5, H6, H7, and H8 were accepted. That way, PEOU and PU are proven to have a connecting role between the influence of the independent variables (SQ and IQ) on the dependent variable (JP) so that H9, H10, H11, and H12 are accepted. The nature of PEOU and PU mediation is full mediation because the direct effect of SQ and IQ on JP is insignificant.

**Table 4:** Hypothesis test results.

Effect(s)		Original Sample	T- Statistics	P-Value	Result
H1	System Quality (X1) ➝ Job Performance (Y)	0.095	1.81	0.07	Not significant
H2	Information Quality (X2) ➝ Job Performance (Y)	0.10	1.44	0.10	Not significant
H3	Perceived Ease of Use (Z1) ➝ Job Performance (Y)	0.26	6.21	0.00	Significant
H4	Perceived Usefulness (Z2) ➝ Job Performance (Y)	0.33	5.65	0.00	Significant
H5	System Quality (X1) ➝ Perceived Ease of Use (Z1)	0.20	3.68	0.00	Significant
H6	Information Quality (X2) ➝ Perceived Ease of Use (Z1)	0.33	5.97	0.00	Significant
H7	System Quality (X1) ➝ Perceived Usefulness (Z2)	0.18	3.14	0.00	Significant
H8	Information Quality (X2) ➝ Perceived Usefulness (Z2)	0.41	6.83	0.00	Significant
H9	System Quality (X1) ➝ Perceived Ease of Use (Z1) ➝ Job Performance (Y)	0.07	3.01	0.00	Full Mediation
H10	Information Quality (X2) ➝ Perceived Ease of Use (Z1) ➝ Job Performance (Y)	0.12	4.22	0.00	Full Mediation
H11	System Quality (X1) ➝ Perceived Usefulness (Z2➝ Job Performance (Y)	0.09	2.54	0.01	Full Mediation
H12	Information Quality (X2) ➝ Perceived Usefulness (Z2)➝ Job Performance (Y)	0.19	4.70	0.00	Full Mediation

## Discussion

The results of this study show that the officers’ JP is not significantly affected by SQ and IQ. It means that the quality of information processing from the CDS has an effect on officer performance, but the effect is insignificant. SQ, IQ, and JP appear to be directly and significantly correlated in earlier studies [9, 10, 17]. Despite the high processing and output quality of CDS, not all officer tasks are reliant on its use. In other words, even though they are required to use CDS, officers still prioritize manual tasks like reporting to the Head of the correction and distributing data summaries directly to him. As a result, CDS is not used to its full potential.

Despite the fact that SQ and IQ have not proven to have an impact on the JP of Sumatran correctional TSO, it is known from the results of this study that SQ and IQ can help officers develop positive attitudes toward the use of CDS, particularly PEOU and PU. When the CDS functions properly and there are no issues with the processing of data, officers feel that the effort they put into using the CDS was not in vain; in other words, their experience operating the CDS is regarded favorably. Officers’ success in gathering data and utilizing its features contributes to their confidence in managing the CDS. Furthermore, because of the CDS’s simple and uncomplicated operation, TSO can quickly recall how to use it. The results of this study are thus in line with earlier research showing that SQ can affect system users’ PEOU [12, 20, 28].

Additionally, the officers believe that using the CDS only necessitates minimal effort when the information provided by the CDS complies with the needs and expectations of the officers in carrying out their duties. With such little effort, they were able to gather precise information. A form of IQ benefit to PEOU is also made available by providing information that is in accordance with job requirements in a way that makes it simple to use. It demonstrates how simple it is for the TSO to use the CDS to get the information they require. Additionally, IQ reflects how well users perceive the quality of the information they receive and how quickly and accurately they do so. The findings of this study concur with earlier studies that found IQ can influence system users’ PEOU [12, 20].

Furthermore, when the CDS can run well and there are no obstacles in the information processing process, the Sumatran Correctional TSO feels that using CDS provides benefits to them, that is the completion of their tasks. Officers can do their jobs well by using a system that can run well and without obstacles. The success of information processing by CDS makes officers perceive the use of CDS as a useful process. Thus, this study’s results align with previous research which stated that SQ can affect PU system users [21, 22, 29].

In addition, when CDS can provide information as expected, officers feel that CDS supports work efficiency. Thus, the relevance of the information to the needs of the Sumatran Correctional TSO Officer in real-time also supports proper work completion. This result aligns with previous research which stated that IQ can affect PU system users [12, 22, 28].

This study also found that PEOU and PU could significantly encourage JP of TSO. With officers feeling that CDS is easy to use, officers will prioritize CDS in completing their tasks. CDS is clear in its use so officers can easily operate it. Officers feel that using CDS is simple and manageable, giving officers a good understanding of the service delivery, they perform through CDS. PEOU also encourages officers to be more accepting and utilizing CDS in completing their duties or work because officers feel they can operate CDS. A system with a high PEOU level will minimize the effort the users give to run it, and the performance results they get will exceed the effort. It is related to improving performance when officers can allocate their efforts to other jobs to make their work effective and efficient. Thus, this study’s results align with previous research which stated that PEOU can affect JP system users [8, 18]. However, there is a study that is unable to prove that perceived ease of use of information systems leads to increased user performance [30].

Then, PU made officers work better through the use of CDS. With officers feeling that CDS provides benefits in completing their work, officers will prioritize CDS in completing their tasks. In its use, CDS has succeeded in helping the correctional TSO to be able to minimize the time they carry out their duties so that they can be more productive at work. Thus, the Sumatran Correctional TSO can achieve their work targets by the provisions set by the organization. That way, the service delivery as their main job can be completed more effectively. When the officers feel that the CDS provides benefits in completing their tasks, they will likely have an idea of the expected performance improvement they will receive in the next use of CDS. It encourages the full and primary utilization of HR, ultimately increasing productivity and performance. Thus, this study’s results align with previous research which stated that PU can affect JP system users [8, 19, 28]. Nonetheless, the findings of this study do not support the results of previous research that users’ perceptions of the usefulness of information systems do not strengthen their performance [30].

In the end, with the results stating that SQ and IQ can encourage PEOU and PU, and PEOU and PU affect JP, it can be stated that the influence of SQ and IQ on JP must be completely through the mediator variables. With the CDS being interactive, functioning well, and able to provide information according to the officer’s job with a correct and clear presentation format, the Sumatran TSO will believe that using the CDS only requires a little effort. It will encourage officers to prioritize the use of CDS, which in turn will impact their performance level. In addition, the TSO satisfaction with the CDS’s functionality and the information it produces can encourage their assumption that the CDS can assist officers in completing the work, and the use of CDS can increase the productivity of officers. The benefits that officers receive from CDS will encourage officers to prioritize the use of CDS so it will impact their performance level. Thus, this study’s results align with previous studies which stated that PEOU and PU fully mediate the effect of SQ and IQ on JP system users [16].

## Conclusion

From the test results and the discussion described previously, this study found that SQ and IQ significantly indirectly affect JP through PEOU and PU. Thus, the quality of information processing from the CDS as a system used to complete the work of officers and the suitability of the information produced by the CDS with the wishes or needs of the officers in carrying out their work, cannot directly affect TSO to carry out activities that contribute to the development the technical core of the organization through the use of HR in its work. However, it requires officers’ perception regarding the ease of using CDS without the need to spend effort and the perception of the Sumatran TSO Officer regarding the use of CDS and the benefits officers get from using CDS in their work. Thus, this study shows similar results to research [9, 10, 16, 17] which discusses the mediating role of PEOU and PU on the influence of SQ and IQ on performance.

## Implications

### Theoretical implications

This research contributes knowledge in the organization and human resources field, especially regarding the influence of SQ and IQ against JP with PEOU and PU as mediating variables. Several prior studies concerning the mediating role of PEOU and PU showed mixed results. Thus, this study can strengthen the statement that PEOU and PU act as mediator variables by fully bridging the influence of SQ and IQ on JP. This study also provides an overview of the use of a specific system for public organizations, how it is used and the results on users’ performance. In this case, it can be seen from the high value of PEOU and PU, and JP that using the system, although not voluntary, can still create a good perception of users and ultimately affect their performance.

### Practical implications

The launch and implementation of the CDS would take a lot of time, effort, and money, so this research can help correctional facilities in the Sumatra region learn how the use of CDS affects the performance of their officers. Furthermore, the findings of this study can be evaluated and used as a guide for advancements in the future. The high PEOU, PU, and JP scores of the Sumatran Correctional TSO and the good SQ and IQ scores of the CDS show that the CDS is a good system. However, it should be emphasized once more how important it is to use CDS to support JP, PEOU, and PU TSO in Sumatran correctional facilities. As a result, all correctional institutions on Sumatra need to improve and preserve the positive perception of CDS among their staff members. This can be accomplished by implementing an intensification program for the usage of CDS through an annual, recurring technical guidance program. This is consistent with other research findings that showed officers desired to utilize it but required time to apply CDS [1]. It is crucial to keep in mind that JP is a crucial factor, particularly in organizations tasked with serving the community, and that the fact that there are many more detainees than officers calls for correctional facilities on Sumatra Island to pay closer attention to the performance of their officers. Additionally, this study can serve as a scientific resource for other organizations regarding the significance of System Quality, Information Quality, Perceived Usability, Perceived Ease of Use, and Job Performance for the sustainability of the organization.

### Limitations and suggestions

Concerning the limitations of this study, the population used is only one working group in correctional institutions, namely Technical Support Officers where further researchers can involve all employees in an organization. Future researchers can use the model in this study on the characteristics of different objects, such as the number of samples, the type of organization, and other matters concerning the object to enrich their findings. In addition, future researchers can also conduct research using different analytical techniques and develop more theories, to broaden and deepen knowledge in the field of human resource management.

## Supporting information

S1 Dataset (XLSX)
